# Circulating Activated Platelets in Children With Long COVID: A Case-Controlled Preliminary Observation

**DOI:** 10.1097/INF.0000000000004470

**Published:** 2024-07-15

**Authors:** Danilo Buonsenso, Silvia Sorrentino, Antonietta Ferretti, Rosa Morello, Piero Valentini, Leonardo Di Gennaro, Erica De Candia

**Affiliations:** * From the Department of Woman and Child Health and Public Health, Fondazione Policlinico Universitario A. Gemelli IRCCS, Rome, Italy; † Centro di Salute Globale, Università Cattolica del Sacro Cuore, Rome, Italy; ‡ Dipartimento di Scienze di Laboratorio ed Ematologiche, Policlinico Universitario A. Gemelli IRCCS, Rome, Italy; § Dipartimento di Medicina e Chirurgia Traslazionale, Università Cattolica del Sacro Cuore, Facoltà di Medicina, Rome, Italy.

**Keywords:** Long COVID, children, platelets

## Abstract

We investigated if children with Long COVID (n=14) have activated platelets compared with healthy controls (n=14). Platelet activation and secretion markers were investigated by flow cytometry using MoAbs directed against P-selectin, CD63, and PAC-1 in quiescent platelets and in platelets stimulated with 10-µM adenosine diphosphate and 25-µM protease activated receptor 1-activating peptide. Circulating platelets of patients with Long COVID had significantly increased expression of the activation marker cytometry using MoAbs directed against P-selectin (*P* = 0.019).

## BACKGROUND

Long COVID is a condition characterized by persisting symptoms several weeks after COVID-19. In adults with this condition, hypercoagulability, endotheliopathy, and thromboinflammation with complement dysregulation have been demonstrated and considered pathogenetic mechanisms.^1,2^ In addition, persistent activation of circulating platelets was demonstrated in adults 1 year after COVID,^3^ possibly expression of immune dysregulation and persistent inflammation triggered by the initial infection.^1–3^

About the pediatric population, observational studies have demonstrated that children also can develop Long COVID. A recent state-of-the-art narrative review showed how children can present persistent symptoms from acute SARS-CoV-2 infection (eg, cough, headaches, fatigue, and loss of taste and smell), new symptoms such as dizziness, or exacerbation of underlying conditions, or develop conditions de novo, including postural orthostatic tachycardia syndrome, myalgic encephalomyelitis/chronic fatigue syndrome, autoimmune conditions, and multisystem inflammatory syndrome in children.^4^ Although these symptoms tend usually to improve over time, studies have found that children can suffer from Long COVID for ≥18 months,^4^ therefore having a significant impact on daily life.

Although Long COVID has been documented in children as well, no studies have investigated the pathogenetic mechanisms of the disease in this population. Given the growing evidence of ongoing thromboinflammation in adults with Long COVID,^4^ we performed this study aiming to explore if activated platelets are more expressed in children with Long COVID compared with controls, as a possible indirect sign of endothelial inflammation.

## METHODS

This study is part of a prospective follow-up of children after the first microbiologically confirmed acute SARS-CoV-2 infection (based on polymerase chain reaction on nasopharyngeal swabs), as previously described.^5^ Among these cohorts, Long COVID was defined as the persistence of otherwise unexplained symptoms negatively impacting daily life for at least three months after initial infection.^6^ Children seen in our follow-up study after initial infection and without complaining about ongoing symptoms and returned to usual pre-COVID activities were classified as recovered. Among the cohort of patients with Long COVID, the most severe group of children having persisting symptoms for 6 to 12 months affecting at least 3 different systems was invited to participate in this study, aiming to explore whether platelet activation is present in pediatric Long COVID. Patients who were already taking medications were excluded from the study. Also, children with signs and symptoms suggesting an acute infection (eg, new acute onset of fever) were excluded.

For every patient, an age- and sex-matched healthy control that fully recovered from a previous SARS-CoV-2 infection was invited to participate. Each couple of patients and control was sampled and analyzed the same day. Platelet activation and secretion markers were investigated by flow cytometry using MoAbs directed against P-selectin, CD63, and PAC-1 in quiescent platelets and in platelets stimulated with 10-µM adenosine diphosphate and 25-µM thrombin receptor activating peptide. Mean fluorescence intensity was measured and expressed as a fold increase of the patient samples relative to paired control samples. Results were expressed as median and ranges.

Statistical analysis was performed by GraphPad Prism 6 using Student’s *t* test, and *P* values were calculated based on the 2-tailed test. *P* < 0.05 was considered statistically significant.

The Long COVID cohort was enrolled as part of a larger, prospective, multidisciplinary follow-up study of children with SARS-CoV-2 infection, approved by the local ethics committee (Ethic approval ID4518, Prot0040139/21), and informed consent was provided. Written and informed consent was obtained from parents/caregivers and from children older than 5 years of age according to local guidance of the ethics committees.

## RESULTS

**FIGURE 1.**
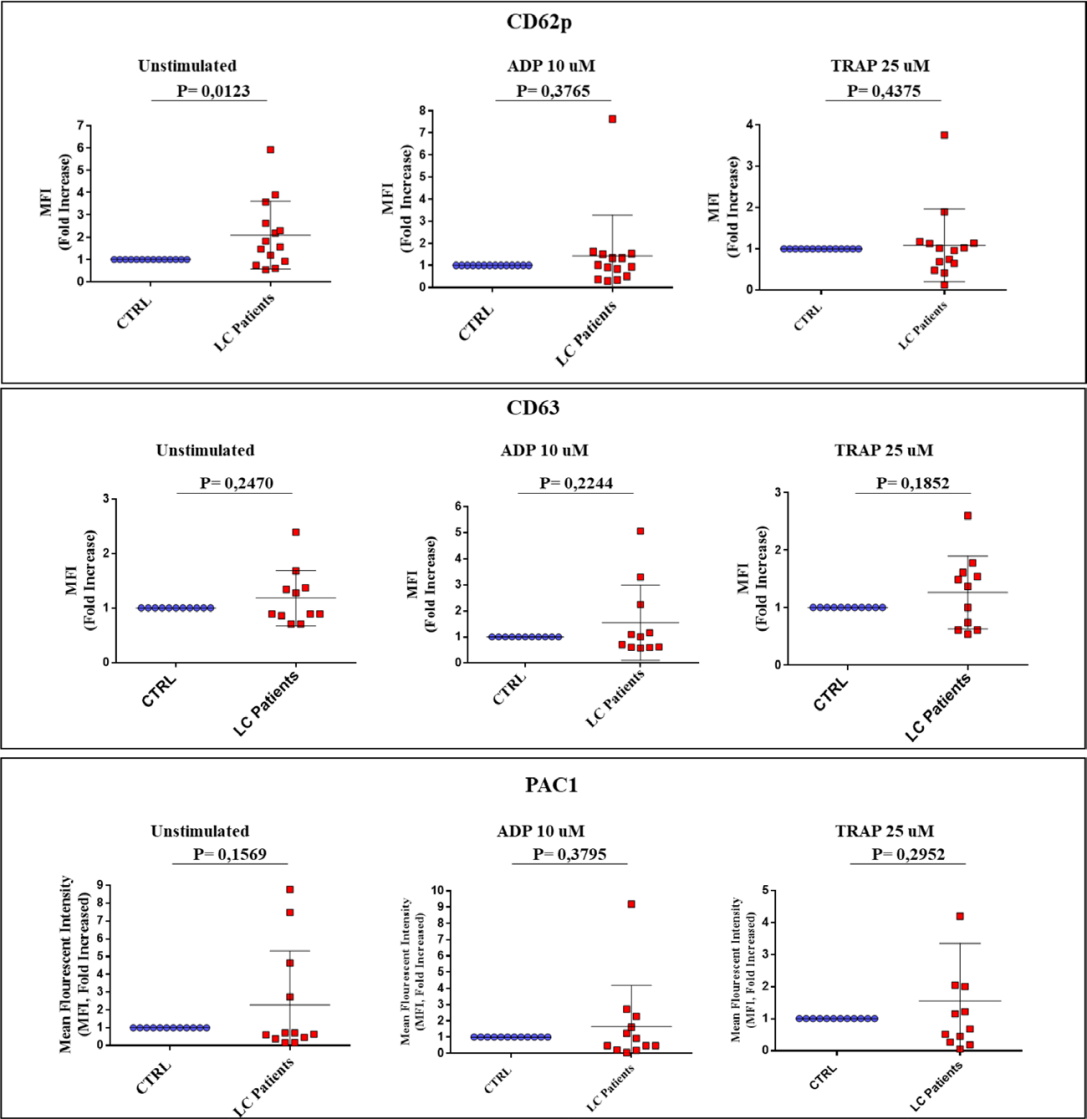
Expression of activation/secretion platelet markers in unstimulated and adenosine diphosphate (ADP)– and thrombin receptor activating peptide (TRAP)–stimulated samples in Long COVID children (patients with LC) and paired controls (CTRL).Sodium-citrated anticoagulated whole blood samples from patients with LC and CTRL were incubated with cytometry using MoAbs directed against P-selectin (CD62p; Clone AK-4, BD Pharmingen), CD63 (Clone CLBGran/12, Beckman Coulter), PAC-1 (Clone PAC-1, BD Pharmingen) MoAbs in resting conditions (unstimulated), or in the presence of 10-µM ADP and 25-µM TRAP stimulating agonists. Each LC patient was investigated in parallel with a control. In each run, the control mean fluorescence intensity (MFI) value was considered the reference (=1) and the paired LC patient MFI was expressed as a fold increase with respect to the paired control value. The results are reported as median±interquartile range. Statistical analysis was performed by Student’s *t* test, and *P* values were calculated based on the 2-tailed test. *P*<0.05 was considered statistically significant.

Fourteen children with ongoing Long COVID and 14 healthy controls were included. Details about demographic and clinical data are provided in Table 1. Fatigue and postexertional malaise were the commonest persisting symptoms. The most frequent combination of persisting symptoms was fatigue plus neurocognitive problems (including headache) and musculoskeletal pains. Circulating platelets of patients with Long COVID had significantly increased expression of cytometry using MoAbs directed against P-selectin compared to controls (Fig. 1A), whereas CD63 and PAC-1 expression in patients with Long COVID were not significantly different compared to controls (Fig. 1D and 1G). After in vitro platelet stimulation by ADP and thrombin receptor activating peptide, all activation and secretion markers were similarly expressed in Long COVID children and controls.

**TABLE 1.** Main Demographic and Clinical Characteristics of the Study Population

	LC N14	Control N14
Age, yr	14.5 (12–18)	15.5 (12–18)
Females	8 (57%)	8 (57%)
Comorbidities Allergy Thyroiditis	3 (12%) 1 2	1 (6.6%) 1
Severity of acute infection		
Asymptomatic	1	2
Mild	13	12
Moderate	0	0
Severe	0	0
Variants*		
Wild	5	4
Alpha	0	1
Delta	1	2
Omicron	8	7
Previously vaccinated (at least 1 dose)	4 (28.6%)	12 (85.7%)
Persisting symptoms		
Fatigue†	10	…
PEM	10	…
Palpitations	7	…
Headache	8	…
Neurocognitive‡	8	…
Musculoskeletal pain	10	…
Gastrointestinal§	5	…
Dysosmia/dysgeusia	1	…
Number of persisting symptoms		
3	5	…
4	3	…
5	4	…
7	2	…
Most common symptom combinations		
Fatigue + neuro/headache+palpitations Fatigue + neuro/headache + musculoskeletal Fatigue + gastrointestinal + palpitations Fatigue + PEM + musculoskeletal Fatigue + PEM + neuro/headache Fatigue + PEM + palpitations	4 7 2 3 4 4	
Duration of persisting symptoms months (min–max)	17.8 (6–24)	…

* Based on the most common circulating variant in Italy at the time of SARS-CoV-2 infection according to the data of the National Ministry of Health, available at https://www.epicentro.iss.it/coronavirus/sars-cov-2-monitoraggio-varianti-rapporti-periodici.

† Based on reporting fatigue “almost always” in the Pediatric Quality of Life Inventory Multidimensional Fatigue Scale (PedsQL Multidimensional Fatigue Scale) in the appropriate sections according to the core outcomes set for pediatric Long COVID.^7^

‡ Neurocognitive problems: brain fog, difficulty concentrating, unusual behavior, and unusual memory problems.

§ Gastrointestinal: abdominal pain, frequent diarrhea, and new onset episodes of reflux.

PEM indicates postexertional malaise.

## DISCUSSION

These data demonstrate that platelets in children with Long COVID circulate in an activated state, as shown by increased expression of P-selectin, a sensitive platelet activation marker, on their membrane. The small number of patients investigated may account for the lack of PAC-1 and CD63 significant increased expression on platelets from Long COVID children, given that these markers could be found increased in 5/14 and 6/14, respectively, of investigated patients. Together with our previous findings of higher d-dimer levels in a subgroup of children with Long COVID,^8^ these data support the hypothesis that persisting chronic inflammation causing coagulation and platelet activation (in addition to endothelial activation) may play a role in children too, similarly to adults. Hence, our results confirm previous findings of activation of hemostasis and inflammation processes in Long COVID children^8,9^ and add the novel finding of platelet activation in these patients. Factors that trigger the activation of platelets in these patients are still unknown although adult studies suggested that viral persistence and endothelial inflammation can be stimulating factors.^1–3^ Recently, viral persistence in megakaryocytes and platelets has been demonstrated, and it has been suggested as a mechanism of platelet activation.^10^ Platelet activation may both contribute to endothelial dysfunction or represent the expression of a systemic inflammatory condition involving endothelial, blood cells, and coagulation in the process of thromboinflammation. In fact, the role of platelets in inflammatory responses is increasingly recognized.^11^ It must be highlighted that activated platelets were found in subjects that were infected during different waves, including omicron. Of note, microvascular damage has also been demonstrated during the acute phases and follow-up of children with multisystem inflammatory syndrome, another postacute complication of pediatric SARS-CoV-2 infection.^12^

Our findings, along with those on multisystem inflammatory syndrome showing microvascular damage, suggest that SARS-CoV-2 infection can determine long-term endothelial inflammation in children similarly to adults. Noteworthy, the present data agree with the increased p-selectin and CD63 expression on circulating platelets of adults 1 year after COVID-19.^3^

We report for the first time that platelets circulate in an activated state in Long COVID children. Adding information to the pathogenesis of Long COVID syndrome may help identification of therapeutic targets. In this regard, some authors have suggested that the use of antithrombotic treatments, such as anticoagulants or antiplatelet drugs, may reverse symptoms.^13–16^ However, despite that our findings may reinforce this theory, we believe that caution is needed for the use of antithrombotic treatment in this context and that appropriate ad hoc studies are needed to confirm such an approach. Larger studies are required to replicate our findings and understand whether specific symptoms correlate with higher platelet activation markers in children.

Nevertheless, these data may have potential implications if confirmed on larger cohorts, both from diagnostic and therapeutic perspectives, as they open to potential novel treatment options for children with prolonged symptoms after SARS-COV2, for example, using drugs with pleiotropic effects on endothelial cells.

We acknowledge that the small number of children represents the main limitation of our study, due to the late start of the study, in 2023, and to choose to recruit only the more severe cases that had not received previous treatments. Also, markers of activated platelets were not elevated in all patients, suggesting that these events may explain only partially why some patients develop Long COVID. More extensive studies investigating further mechanisms involved in the pathogenesis of the disease are needed for children with Long COVID.

## ACKNOWLEDGMENTS

*This study is part of a larger study funded by Pfizer to Dr Buonsenso. The funding body had no role in study design nor in data interpretation. Dr Buonsenso has received funds to study Long COVID from Pfizer and Roche*.
